# Exposure Levels of Pyrethroids, Chlorpyrifos and Glyphosate in EU—An Overview of Human Biomonitoring Studies Published since 2000

**DOI:** 10.3390/toxics10120789

**Published:** 2022-12-15

**Authors:** Helle Raun Andersen, Loïc Rambaud, Margaux Riou, Jurgen Buekers, Sylvie Remy, Tamar Berman, Eva Govarts

**Affiliations:** 1 Clinical Pharmacology, Pharmacy and Environmental Medicine, Department of Public Health, University of Southern Denmark (SDU), 5000 Odense, Denmark; 2 Santé Publique France, Environmental and Occupational Health Division, 94410 Saint-Maurice, France; 3 VITO Health, Flemish Institute for Technological Research (VITO), 2400 Mol, Belgium; 4 Israel Ministry of Health (MOH-IL), Jerusalem 9446724, Israel

**Keywords:** pyrethroids, chlorpyrifos, glyphosate, human biomonitoring, urinary concentration

## Abstract

Currently used pesticides are rapidly metabolised and excreted, primarily in urine, and urinary concentrations of pesticides/metabolites are therefore useful biomarkers for the integrated exposure from all sources. Pyrethroid insecticides, the organophosphate insecticide chlorpyrifos, and the herbicide glyphosate, were among the prioritised substances in the HBM4EU project and comparable human biomonitoring (HBM)-data were obtained from the HBM4EU Aligned Studies. The aim of this review was to supplement these data by presenting additional HBM studies of the priority pesticides across the HBM4EU partner countries published since 2000. We identified relevant studies (44 for pyrethroids, 23 for chlorpyrifos, 24 for glyphosate) by literature search using PubMed and Web of Science. Most studies were from the Western and Southern part of the EU and data were lacking from more than half of the HBM4EU-partner countries. Many studies were regional with relatively small sample size and few studies address residential and occupational exposure. Variation in urine sampling, analytical methods, and reporting of the HBM-data hampered the comparability of the results across studies. Despite these shortcomings, a widespread exposure to these substances in the general EU population with marked geographical differences was indicated. The findings emphasise the need for harmonisation of methods and reporting in future studies as initiated during HBM4EU.

## 1. Introduction

The general population is exposed to pesticides from residues in food items [1,2], but drifting from agricultural areas, and indoor use of biocides are other important sources of exposure [3,4,5,6]. In addition, some individuals are occupationally exposed. Currently used pesticides are metabolised and excreted, primarily in the urine, within a few days [7,8,9,10,11]. Urinary concentrations of pesticides or their metabolites are therefore useful as biomarkers for the integrated pesticide exposure from all sources. Within the European Human Biomonitoring Initiative (HBM4EU) the following pesticides were prioritised: pyrethroids (whole group), chlorpyrifos and dimethoate (organophosphate insecticides), fipronil (phenyl pyrazole insecticide), and glyphosate (organophosphate herbicide) in combination with polyethoxylated tallow amine (POEA) used as additive in glyphosate herbicide formulations. The prioritisation strategy has been described previously [12]. The primary aim was to get better information on the human internal exposure of these substances in the EU population(s), including potential differences between countries and population groups and time-trends. Another goal was to identify main sources and pathways of exposure across the member states.

Since no suitable urinary biomarkers were available for dimethoate, fipronil or POEA there was no existing European human biomonitoring (HBM)-data on these substances. For the remaining pesticides (pyrethroids, chlorpyrifos and glyphosate) the analytical methods were evaluated and harmonised and used for analysing urine samples collected in HBM4EU Aligned Studies [13,14]. These results have recently been published [14,15,16,17,18] or are in preparation for publication and were not available when the literature searches were completed for this review. The aim of this study was to present additional HBM studies on urinary metabolite concentrations of the priority pesticides: pyrethroids, chlorpyrifos, and glyphosate, across the HBM4EU partner countries published since 2000 in order to supplement the HBM-data obtained from the HBM4EU Aligned Studies. The findings in this review, combined with the results obtained from the HBM4EU-aligned studies, will provide a complete picture of the current HBM-data for these pesticides in Europe and might be useful for the planning of future HBM-studies.

## 2. Materials and Methods

Literature searches were performed in PubMed and Web of Science, for each of the search terms ‘pyrethroid*’, ‘chlorpyrifos OR chlorpyrifos-methyl’, and ‘glyphosate OR AMPA’ combined with ‘urine AND human’ or ‘human biomonitoring’ restricted to publications published between 01.01.2000 and 30.06.2022. We used no language restriction if an English abstract was provided. After exclusion of duplicates, all abstracts (and if necessary, method sections) were screened and only studies that presented HBM data based on urine samples collected in HBM4EU participating countries (i.e., EU Member States, as well as Norway, Iceland, Israel, Switzerland, and UK) were included. Publications with a focus on method development and/or validation were excluded if they presented HBM-data from less than 20 individuals and/or the participants were directly exposed to the pesticides as a part of the study.

Table 1 presents the urinary pesticide biomarkers included. Data on limit of detection/quantification (LOD/LOQ), the frequency of detection (% above LOD/LOQ) and urine concentrations for the biomarkers were extracted. To make the studies as comparable as possible, the concentrations are presented as 50th percentiles (P50 or medians) and 95th percentiles (P95) in micrograms per litre (μg/L). Volume based concentrations were chosen because these values were provided in most studies and dilution adjustments (creatinine, osmolarity, relative density) varied between studies. If these data were not available other measures of central and upper concentrations were used as indicated in Tables. We also extracted information on some population characteristics and urine sampling method. Publications were not assessed for their overall quality but some information on quality control of the analytical methods was extracted, as was information on the analytical platform and deconjugation procedure, i.e., enzymatic hydrolyses (β-glucuronidase and/or sulfatase) or acidic hydrolysis, to help assess comparability of the urinary concentration between the studies. Finally, potential information on exposure determinants identified within the individual studies was included.

## 3. Results

### 3.1. Pyrethroids

We identified 44 studies presenting urinary pyrethroid metabolite concentrations in European populations from 13 different countries (Table 2) mainly from the Western and Southern part of the EU. The datasets represent a range of different population groups including pregnant women (5 studies) and children (12 studies). Most of the studies were regional and only three studies claimed to be nationally representative for the respective population group, i.e., for pregnant women [19] and adults [20] in France and for children in Germany [21]. The majority of studies were based on single spot urine samples (25 studies) or first morning voids (FMV) (12 studies).

The generic pyrethroid metabolite, 3-PBA, representing the combined exposure to most pyrethroids, was included in most studies, while inclusion of more specific metabolites was more variable and only three studies included CFCA which is a metabolite of bifenthrin and λ-cyhalothrin [22,23,24]. The specific metabolites are formed in parallel to 3-PBA by ester cleavage of the parent compounds catalysed by carboxylesterase enzymes [25]. Accordingly, most studies showed highest detection frequency and urine concentration of 3-PBA.

The methodologies used to determine the metabolite concentrations varied both regarding pre-treatment of the urine sample and the analytical platform used for detection. Pyrethroid metabolites are present mainly as phase II conjugates (mainly glucuronide) in urine [26] and omitting a deconjugation step might underestimate the exposure level. Therefore, information on a deconjugation step or not, as well as the method used (i.e., enzymatic or acidic hydrolyses) were reported for each study (Table 2). Acidic hydrolyses is less specific than enzymatic deconjugation leading to potential release from other conjugates than glucuronide and thus slightly higher concentrations, as previously demonstrated for 3-PBA [27]. Of the included studies, 18 reported enzymatic deconjugation using β-glucuronidase, 18 reported acidic hydrolysis, and 8 studies did not mention any deconjugation step. For these studies, it was not possible to determine if the description was missing or deconjugation was not performed. Two of these datasets, based on the French PELAGIE cohort [28,29], and one from the Spanish INMA-Granada cohort [30] reported very low 3-PBA concentrations compared to the other studies. Thus, we suspect that these concentrations might be underestimated. The potential impact on the concentrations of the more specific pyrethroid metabolites is difficult to assess since these concentrations were more like those in other datasets.

Regarding the analytical platform, LC-MS/MS was used for all the metabolites in 19 studies. Two datasets from children and mothers from the PELAGIE cohort, respectively, used LC only for 3-PBA and 4-F-3-PBA while other metabolites were analysed by GC-MS/MS [28,29]. GC-MS/MS was used for all metabolites in 21 of the studies. One study used an immunoassay [31] and one study did not describe the analytical method but referred to an ISO9001 accredited lab [22]. Reported LOD/LOQs for, e.g., 3-PBA varied from a LOD of 0.004 to a LOQ of 0.8 µg/L between the included studies leading to large variation in detection frequencies.

The wide variation in urine sampling and analytical methods hamper the comparability of the results across studies and the possibility to assess time trends and geographical differences in pyrethroid exposure. Two studies from Sweden reported increasing 3-PBA concentrations between 2000 and 2017 among young adults [23] and between 2009 and 2014 among women after delivery [32]. Further, the highest 3-PBA concentrations in general population groups were reported from studies with urine samples collected after 2015, i.e., among children from Cyprus (median 1.93 µg/L) [33], the Valencia region in Spain for children (1.63 µg/L) [34] and lactating mothers (1.7 µg/L) [35] and among adolescents (0.87 µg/L) and children (0.98 µg/L) from Flanders in Belgium [36,37].

When urine samples were obtained from both children and adults within the same country and time period, the 3-PBA concentrations were in general higher in children than adults, e.g., medians of 0.56 vs. 0.24 µg/L, respectively, in Denmark [38,39], 0.29 vs. 0.23 µg/L in Poland [40], and 0.40 vs. 0.24 µg/L in Slovenia [41].

A limited number of the studies investigated exposure sources for pyrethroids in the general population. Regarding dietary exposure determinants, consumption of vegetables, fruits, and food items based on cereals (e.g., pasta and whole grain bread) [20,28,42,43,44,45] and in some studies also fish [19,20] was associated with higher urinary pyrethroid metabolite concentrations. High organic food consumption was associated with lower urinary concentrations [2,28].

For non-dietary exposure determinants, indoor use of biocides [19,37,43,44,45,46] including pet care products [40] was related to higher urinary metabolite concentrations. Two studies from France reported higher pyrethroid metabolite concentrations among pregnant women [19] and children [28] living in the vicinity of cultivated crops indicating some drift-exposure of residents in agricultural areas. Further, higher concentrations were reported from children and parents in rural areas in Poland compared to urban residence [40] and among children whose parents were occupationally exposed to pesticides [28,38]. Only few, and mostly small, studies included occupationally exposed groups such as pesticide applicators [47], farmers [31,48,49] and greenhouse workers [50] or focused on residents after indoor use of pyrethroids [51,52]. Overall, these studies found higher urinary concentrations of pyrethroid metabolites related to recent occupational or residential exposure although a few studies were unable to demonstrate a difference, likely because of high LODs and correspondingly low numbers of participants with detectable concentrations [48,52].

**Table 2:** Human biomonitoring studies of pyrethroid exposure based on urine samples from European populations.

Country, Region, (Cohort)	Study Population	Sampling Year and Method	Analytical Method and Quality Control (QC), Correction Method for Urine Dilution	LOD/LOQ, DF, and Urinary Concentrations (μg/L Unless Other Stated)		Exposure Determinants Reported	Ref
*Northern EU*				**3-PBA**	**Specific metabolites**		
Denmark, Funen (OCC)	Pregnant women (*n* = 948)	2010–2012; Spot urine (morning after overnight fasting), GW 28	Enzymatic hydrolyses; LC-MS/MS; QC: Isotope-labelled internal standards, participation in G-EQUAS for 3-PBA, *trans*-DCCA, and *cis*-DBCA; Creatinine	LOD: 0.03 DF: 94.3 P50: 0.24 P75: 0.46	4F-3PBA LOD: 0.2 DF: 0.1 *trans*-DCCA LOD: 0.4 DF: 11.4 *cis*-DCCA LOD: 0.5 DF: 2.8 *cis*-DBCA LOD: 0.5 DF: 3.0	3-PBA not significantly related to any demographic characteristics, no information on diet or home use of pesticides	Dalsager et al., 2019 [39]
Denmark, Funen (DGCC)	Children, 10–16 y (*n* = 143)	2011–2012; Spot urine	Enzymatic hydrolyses; LC-MS/MS; QC: Isotope-labelled internal standards, participation in G-EQUAS for 3- PBA, *trans*-DCCA, and *cis*-DBCA; Creatinine	LOD: 0.03 DF: 100 P50: 0.56 P95: 8.90	*trans*-DCCA LOD: 0.4 DF: 9.8 P95: 2.3 *cis*-DCCA LOD: 0.5 DF: 2.8 *cis*-DBCA LOD: 0.5 DF: 2.1	Higher 3-PBA in samples from autumn/winter than spring/summer, and if mother were occupationally exposed to pesticides	Andersen et al., 2021 [38]
Sweden, Scania	Adolescents aged 17–21 (approx. 200 per year in 2000, 2004, 2009, 2013, and 2017)	2000–2017; Spot urine	Enzymatic hydrolyses; LC-MS/MS; QC: spiked urine samples, participate in Erlangen inter-laboratory comparison for 3-PBA; Urine density and creatinine	LOD: 0.009 DF: 96–100 P50: 0.11–0.21 P95: 0.56–0.92	4F-3PBA LOD: 0.005 DF: 42–74 P50: <LOD–0.01 P95: 0.02–0.06 CFCA LOD: 0.006 DF: 39–90 P50: <LOD–0.02 P95: 0.05–0.40 DCCA LOD: 0.017 DF: 97–99 P50: 0.16–0.22 P95: 0.53–0.89	Increasing temporal trend for 3-PBA (3.7% per year), no information on exposure sources	Noren et al., 2020 [23]
Sweden, Uppsala County (POPUP)	Women, after delivery (*n* = 178)	2009–2014; Morning spot urine, 3 weeks after delivery	Enzymatic hydrolyses; LC-MS/MS; QC: Isotope-labelled internal standards, participate in Erlangen inter-laboratory comparison; Urine density	LOD: 0.03 DF: 98 P50: 0.22 Max: 2.59	NR	Increasing trend in 3-PBA from 2009 to 2014, no information on determinants	Gyllen-hammar et al., 2017 [32]
Sweden, Värmland county (SELMA)	Pregnant women (*n* = 718)	2007–2010; FMV, GW 10	Enzymatic hydrolyses; LC-MS/MS; QC: Isotope-labelled internal standards; Creatinine	LOD: 0.017 DF: 99 GM (GSD) 0.16 (2.7)	NR	NR	Tanner et al., 2020 [53]
*Western EU*							
Belgium, Flanders, FLEHS IV	Adolescents, 14–15 y (*n* = 415)	2017–2018, spot urine	Enzymatic hydrolyses; LC-MS/MS; QC: Isotope-labelled internal standards, participation in G-EQUAS for 3-PBA; Specific gravity	LOD: 0.03 DF: 99.5 P50: 0.87 P90: 2.77	NR	Higher 3-PBA associated with higher household education in binary analyses	Schoeters et al., 2022 [36]
Belgium, Walloon region	Children, 9–12 y (*n* = 258) from five different locations (urban or agricultural)	2016; FMV	Enzymatic hydrolyses; GC-MS/MS; QC: Internal standards and materials from previous G-EQUAS programs; Creatinine	LOQ: 0.09 DF: 99.6 P50: 0.98 P95: 5.33	4F-3PBA LOQ: 0.11 DF: 2.2 *trans*-DCCA LOQ: 0.15 DF: 93.2 P50: 0.66 P95: 4.29 *cis*-DCCA LOQ: 0.50 DF: 40.3 P95: 2.01	Higher *trans*-DCCA and 3-PBA associated with indoor use of pyrethroids, and negatively associated with consumption of grey bread (graubrot)	Pirard et al., 2020 [37]
France, NutriNet-Sante	Adults from general population, mean age 58.5 y (*n* = 300, divided in two matched groups based on low (<10%) or high (>50%) organic food consumption from questionnaire	2014; Spot urine, fasted 6 h before collection	Samples were analysed both without and with a deconjugation step included. Enzymatic hydrolyses: LC-MS-MS; QC: internal standards and control samples; Creatinine	LOD: 0.02 Organic DF: 23 Mean: 0.12 Conventional DF: 35 Mean: 0.13	4F-3PBA LOD: 0.02 Organic: DF: 3 Mean: 0.012 Conventional: DF: 3 Mean: 0.014	3-PBA was slightly lower if high organic food consumption but only significant when urine samples were analysed without a deconjugation step (mean: 0.026 vs. 0.042 μg/L for high and low organic intake)	Baudry et al., 2019 [2]
France (ELFE)	Pregnant women (*n* = 1077), nationally representative	2011; Spot urine, at delivery	Enzymatic hydrolyses; GC-MS/MS; QC: Internal standards and control samples; Creatinine	LOD: 0.004 DF: 100 P50: 0.36 P95: 1.89	4F-3PBA LOD: 0.005 DF: 17.8 P95: 0.02 *trans*-DCCA LOD: 0.006 DF: 100 P50: 0.26 P95: 2.29 *cis*-DCCA LOD: 0.003 DF: 100 P50: 0.16 P95: 0.91 *cis*-DBCA LOD: 0.005 DF: 100 P50: 0.23 P95: 1.38	Urinary concentrations of pyrethroid metabolites (3-PBA or sum of metabolites) were positively related to smoking during pregnancy, consuming of fish and alcohol, domestic pesticide use and living in the vicinity of crops during pregnancy.	Dereu-meaux et al., 2018, 2016 [19,46]
France, Brittany (PELAGIE)	Children, 6 y, (*n* = 245), 55 % rural residence	2009–2012; FMV	No information on deconjugation; LC-MS/MS for 3-PBA and 4-F-3PBA GC-MS/MS for *Trans*-DCCA, *Cis*-DCCA, and *Cis*-DBCA; QC: Internal standards; Creatinine	LOD: 0.008 DF: 63 P50: 0.02 P95: 0.20	4F-3PBA LOD: 0.003 DF: 15.8 P95: 0.02 *trans*-DCCA LOD: 0.01 DF: 95 P50: 0.22 P95: 1.75 *cis*-DCCA LOD: 0.07 DF: 64 P50: 0.09 P95:0.49 *cis-*DBCA LOD: 0.07 DF: 84 P50: 0.20 P95: 1.12	3-PBA and *Cis*-DBCA higher in children living in proximity (<500 m) to crops. 3-PBA correlated to high fruit consumption, parental occupational pesticide exposure. *cis*-DBCA related to high consumption of cereal and whole grain bread. Higher *trans/cis*-DCCA when floor cleaning at least twice a week; *cis*-DCCA associated with daily consumption of pasta, rice, or semolina. Organic food intake was associated with lower 3-PBA, *cis*-DBCA and *trans*-DCCA.	Gloren-nec et al., 2017 [28]
France, Brittany (PELAGIE)	Pregnant women (*n* = 205), 55.1% rural residence	2002–2006; FMV, GW 6–19	No information on deconjugation; LC-MS/MS for 3-PBA and 4-F-3PBA, GC-MS/MS for *trans*- and *cis*-DCCA, and *cis*-DBCA; QC: Internal standards; Creatinine	LOD: 0.008 DF: 30.2 P90: 0.075	4F-3PBA LOD: 0.003 DF: 8.8 *trans*-DCCA LOD: 0.01 DF: 98 P50: 0.14 P90: 0.57 *cis*-DCCA LOD: 0.07 DF: 64.9 P50: 0.09 P90: 0.30 *cis*-DBCA LOD: 0.07 DF: 68.3 P50: 0.11 P90: 0.39	NR	Viel et al., 2017, 2015 [29,54]
France (Pilot-ELFE study)	Pregnant women (*n* = 93)	2007; Spot urine at delivery	Acidic hydrolysis; GC-MS/MS: QC: Isotope-labelled internal standards; Creatinine	LOD: 0.046 DF: 100 P50: 0.37 P95: 3.06	4F-3PBA LOD: 0.1 DF: 2 DCCA LOD: 0.04 DF: 100 P50: 0.34 P95: 4.13 CFCA LOD: 0.004 DF: 18 P95: 0.15 *cis*-DBCA LOD: 0.02 DF: 86 P50: 0.13 P95: 0.54	NR, method validation study	Hardy et al., 2021 [55]
France (ENNS)	Adults, 18–74 y (*n* = 396), nationally representative	2006–2007; FMV	Acidic hydrolysis/; GC- MS/MS; QC: Internal standards and quality control samples; Creatinine	LOD: 0.03 DF: 98.1 P50: 0.65 P95: 4.36	4F-3PBA LOD: 0.03 DF: 29.8 P95: 0.82 *trans*-DCCA LOD: 0.03 DF: 86.1 P50: 0.31 P95: 3.85 *cis*-DCCA LOD: 0.03 DF: 56.1 P50: 0.13 P95: 1.42 *cis*-DBCA LOD: 0.03 DF: 83.1 P50: 0.36 P95:2.33	Associations with higher intake of solanaceous (e.g., tomatoes, aubergines) vegetables and shellfish and non-significantly with fish intake.	Fréry et al., 2017 [20]
France, Limousine region	Adults, 24–62 y, (*n* = 39)	No information on sampling year; Spot urine	Acidic hydrolysis; LC-MS/MS; QC: Isotope-labelled internal standards; Creatinine	LOD: 0.015 DF: 100 P50: 0.63 P95: 2.05	LOD: 0.015 all metabolites 4F-3PBA DF: 10 P95: NR *trans*-DCCA DF: 100 P50: 0.33 P95: 1.10 *cis*-DCCA DF: 97 P50: 0.19 P95: 0.49 *cis*-DBCA DF: 97 P50: 0.18 P95: 0.69	NR, method development study	Le Grand et al., 2012 [56]
Germany	Adults, 26–58 y (*n* = 38)	2012; Spot urine	Acidic hydrolysis; GC-MS/MS; QC: Isotope-labelled internal standards; Creatinine	LOQ: 0.01 DF: 100 P50: 0.22 P95: 1.79	LOQ: 0.01 all metabolites 4F-3PBA DF: 5 *trans*-DCCA DF: 100 P50: 0.17 P95: 0.92 *cis*-DCCA DF: 100 P50: 0.08 P95: 0.57 *cis*-DBCA DF: 80 P50: 0.04 P95: 0.28 CFCA DF: 90 P50: 0.04 P95: 0.98	NR, method development study	Schettgen et al., 2016 [24]
Germany (GerES IV)	Children, 3–14 y (*n* = 598), nationally representative	2003–2006; Spot urine	NR but references to previous GerES-studies	LOQ: 0.1 DF: 98 P50: 0.43 P95: 3.80	LOQ: 0.01 for all metabolites *trans*-DCCA DF: 86 P50: 0.25 P95: 2.46 *cis*-DCCA DF: 60 P50: 0.12 P95: 1.00	Girls had higher concentrations than boys, no information on determinants	Schulz et al., 2009 [21]
Germany (GerES IV), pilot study	Children, 2–17 y (*n* = 396), Berlin and two rural areas	2001–2002; Morning spot urine	No information on deconjugation; GC-MS; Internal standards and participation in G-EQUAS	LOQ: 0.1 DF: 90 P50: 0.29 P95: 2.35	LOQ: 0.01 for all metabolites 4F-3PBA DF: <1 *trans*-DCCA DF: 74 P50: 0.19 P95: 1.73 *cis*-DCCA DF: 56 P50: 0.11 P95: 0.74 *cis*-DBCA DF: 22 P95: 0.52	3-PBA, *cis*- and *trans*-DCCA negatively associated with child age and positively with permethrin in house dust, use of biocides indoor, consumption of boiled vegetables, and Berlin sampling area	Becker et al., 2006 [43]
Germany	Occupational exposure: male workers exposed to pesticides in agriculture (*n* = 19), pest control (*n* = 15) or greenhouses (*n* = 2)	Sampling year is unclear; 24 h urine collected after pyrethroid application, repeated sampling for some workers	Acidic hydrolysis; GC-MS; Internal standards; Creatinine	LOD: 0.5 DF: 67–100 P50: 0.6–2.9 μg/g crea	4F-3PBA LOD: 0.5 DF: 0–3 DCCA LOD: 0.5 DF: 46–100 P50: <LOD–2.9 μg/g crea *cis*-DBCA LOD: 0.3 DF: 0–71 P50: <LOD–0.50 μg/g crea	Highest concentration of 3-PBA and DCCA in pest control workers.	Hardt and Angerer 2003 [47]
Germany, Frankfurt am Main	General population, 0–65 y (*n* = 1177), urban residence	1998; Spot urine	Acidic hydrolysis; GS-MS; QC: internal standards and participation in G-EQUAS; Creatinine	NR	LOD: 0.1–0.2 for all metabolites 4F-3PBA DF: 16.4 P95: 0.27 *trans*-DCCA DF: 65.3 P50: 0.24 P95:1.43 *cis*-DCCA DF: 29.4 P95: 0.51 *cis*-DBCA DF: 19.3 P95: 0.30	No significant correlation with age, smoking habits, sampling season or permethrin in dust	Heudorf and Angerer 2001 [57], Schettgen et al., 2002 [58]
Germany, Frankfurt am Main	Children 0–17.9 y, (*n* = 673), urban residence	1998; Spot urine	Acidic hydrolysis; GC-MS; QC: internal standards and participation in G-EQUAS; Creatinine	NR	LOD 0.1–0.2 for all metabolites, DFs not provided 4F-3PBA P95: 0.30 *trans*-DCCA P50: 0.25 P95: 1.22 *cis*-DCCA P95: 0.44 *cis*-DBCA P95: 0.30	No correlation with age of the children or permethrin in dust	Heudorf et al., 2004 [59]
Germany	Residents after pyrethroid used indoors, (*n* = 56)	1996–1998; collected before and day 1 and 3, and 4–6 months and 10–12 months after application, Spot and 24 h urine	Acidic hydrolysis; GC-MS; QC: internal standards; Creatinine	LOD: 0.2 DF: 5–28 P95: 0.2–1.8	4F-3PBA DF: 0–5 P95: <LOD–LOD *trans*-DCCA DF: 4–32 P95: <LOD–1.5 *cis*-DCCA DF: 3–21 P95: >LOD–0.6 *cis*-DBCA DF: 0–6 P95: <LOD–0.3	Highest concentrations seen 1 and 3 days after application but most samples were below LOD	Leng et al., 2003 [51]
Germany, Hannover	Residents after pyrethroid (permethrin) used indoors in homes with carpets of wool, 9 months–78 y (*n* = 145),	1996–1998, 24 h urine from adults, spot urine from young children	Method not described but reference to Leng et al., 1997 (se study above)	LOD: 0.2 DF: 28 P95: 0.90	DCCA LOD: 0.2 DF: 19 P95: 1.50	Most samples below LOD, children tended to have higher detection frequency than adults although not statistically significant (few children included)	Berger-Preiss et al., 2002 [52]
UK	Adults, 63.8 ± 10.4 y (*n* = 111, representing 65 twin pairs from the TwinsUK-cohort)	No information	Enzymatic hydrolyses; LC-MS/MS; QC: Isotope-labelled internal standards	LOD: 0.015 DF: 80 P50: 0.12 P75: 1.8	4F-3PBA LOD: 0.015 DF: 10 *trans*-DCCA LOD: 0.02 DF: 96.9 P50: 0.18 P75: 1.2 *cis*-DCCA LOD: 0.01 DF: 98.4 P50: 0.07 P75: 0.38 *cis*-DBCA LOD: 0.015 DF: 95.4 P50: 0.08 P75: 0.42	No difference between urban or rural residence, not possible to investigate impact of organic food as planned because of few participants with high intake of organic food	Mesnage et al., 2022 [60]
UK, Lothian, Kent and Norfolk	Farmers and residents in agricultural areas, <100 m from sprayed fields, Adults >18 y (*n* = 238) and children 4–12 y (*n* = 58); 140 with repeated samples	2011–2012; FMV within 2 days after spraying events and outwith the spraying season (140 with repeated samples)	Enzymatic hydrolyses; LC-MS/MS; QC: Participate in G-EQUAS; Creatinine		*cis/trans*-DCCA LOD: 1.0 DF: 7 Max conc (μg/g crea): Outwith spraying season: 15.4 Within spraying season, 10.8 After spray event: 7.0	No difference related to spraying activity or spraying season but low detection frequency (high LOD)	Galea et al., 2015 [48]
UK,	Randomly sought adult volunteers, >18 y (*n* = 405), nationwide	NI on sampling year (after 2005); Spot urine	No description but all analyses were carried out by an ISO9001: 2008 accredited laboratory with internal quality control, participation in G-EQUAS; Creatinine	LOD: 0.5 nM DF: 87 P95: 6.1	LOD: 0.5 nM for all metabolites *trans*-DCCA DF: 66 P95: 1.6 *cis*-DCCA DF: 54 P95: 0.8 *cis*-DBCA DF: 50 P95: 1.6 CFCA DF: 41 P95: 3.2	NR	Bevan et al., 2013 [22]
*Eastern EU*							
Poland, Łódź	Women, 25–45 y (*n* = 450) attending a fertility clinic,	2017–2019; 1–2 spot urine per IVF cycle (total 739 urine samples)	No information on deconjugation; GC–MS; QC: No information; Specific gravity	LOD: 0.1 DF: 68 GM: 0.35 GSD: 2.66	LOD: 0.1 for all metabolites *trans*-DCCA DF: 45 GM: 0.43 GSD: 2.48 *cis*-DCCA DF: 34 GM: 0.29 GSD: 2.18 *cis*-DBCA DF: 22 GM: 0.28 GSD: 2.51	NR	Radwan et al., 2022 [61]
Poland Łódź	Young men, 19–33 y (*n* = 306), urban area residence	2015–2018; morning spot urine	Acidic hydrolysis; GC-MS; QC: Internal standards (spiked urine samples); participation in G-EQUAS; Specific gravity	LOD: 0.1 DF: 69 P50: 0.20 P95: 1.67	LOD: 0.1 for all metabolites *trans*-DCCA DF: 76 P50: 0.26 P95: 2.07 *cis*-DCCA DF: 36 P95: 0.94 *cis*-DBCA DF: 32 P95: 0.93	*trans*-DCCA and 3-PBA associated with dog ownership, pesticide use indoor and household pets. Seeds and nuts consumption was also associated with higher 3-PBA and vegetable juice intake with higher *trans*-DCCA.	Rodzaj et al., 2021 [45]
Poland	Women, 25–39 y, (*n* = 511), attending a fertility clinic	No information on sampling year (likely after funding grant in 2017); Spot urine	Acidic hydrolysis; GC–MS; QC: Isotope-labelled internal standards and participation in G-EQUAS; Specific gravity	LOD: 0.1 DF: 66.5 P50: 0.25 P95: 2.28	LOD: 0.1 for all metabolites *trans*-DCCA DF: 34.9 P95: 3.47 *cis*-DCCA DF: 32.8 P95: 1.54 *cis*-DBCA DF: 19.4 P95: 2.17	NR	Jurewicz et al., 2020 [62]
Poland, Gdansk,	General population, 5–77 y (*n* = 132), Urban residence	2010–2011; FMV	Acidic hydrolysis; GC-MS; QC: Internal standards; Creatinine	LOD: 0.1 DF: 80 P50: 0.26 P95: 1.15	LOD: 0.1 for all metabolites *trans*-DCCA DF: 7 P95: 0.12 *cis*-DCCA DF: 8 P95: 0.15 *cis*-DBCA DF: 11 P95: 0.31	No age or sex related differences in 3-PBA	Wielgo-mas et al., 2013 [63]
Poland, North	Children <18 y (*n* = 184) and parents (*n* = 190); Urban or rural residence	2012; FMV	Acidic hydrolysis; GC-MS; QC: Internal standards, participation in G-EQUAS; Creatinine	LOD: 0.1 DF: 82.4 P50: 0.25 (all); 0.29 (children); 0.23 (adults) P95: 1.24 (all)	LOD: 0.1 for all metabolites *trans*-DCCA DF: 46.8 P95: 1.00 *cis*-DCCA DF: 46 P95: 0.89 *cis*-DBCA DF: 17.1 P95: 0.50	Higher concentrations of all metabolites in rural areas, higher 3-PBA in participants using pesticide containing pet care products in the last 6 months for both rural and urban locations. Higher 3-PBA in children than adults	Wielgo-mas and Piskuno-wicz 2013 [40]
Poland, Łódź	Adult men, 23–45 y (*n* = 195) recruited from fertility clinic	2008–2011; Spot urine	Acidic hydrolysis; GC-MS; QC: participation in G-EQUAS; Creatinine	LOD: 0.1 DF: 71.6 P50: 0.16 P95: 0.50	LOD: 0.1 for all metabolites *Trans*-DCCA DF: 65.5 P50: 0.16 P95: 0.62 *cis*-DCCA DF: 58 P50: 0.12 P95: 0.46 *cis*-DBCA DF: 16.8 P95: 0.27	NR	Radwan et al., 2015, Jurewicz et al., 2016 [64,65]
Slovenia, Ljubljana (PHIME)	Children 7–8 y (*n* = 168) and their mothers (*n* = 168)	2016; Spot urine	Enzymatic hydrolyses; UPLC-MS/MS; QC: Isotope-labelled internal standards and participation in G-EQUAS; Specific gravity and creatinine	LOD: 0.018 Children DF: 80 P50: 0.40 Max: 12.0 Mothers DF: 76 P50: 0.24 Max: 12.0	4F-3PBA LOD: 0.019 Children DF: 30 Max: 0.53 Mothers DF: 16 Max: 0.73	Children had higher concentrations than mothers, no significant associations with demographic variables (education, smoking, BMI etc.)	Bravo et al., 2020 [41]
*Southern EU*							
Cyprus, Limassol (ORGANIKO)	Children, 10–11 y (*n* = 177), urban area	2017; FMV	No information on deconjugation; LC-MS/ MS; QC: Analysed in HBM4EU-accredited lab; Creatinine	LOQ: 0.1 DF: 100 P50: 1.93 P95: 6.59	LOQ: 0.2 for DBCA, 0.1 for all other metabolites 4F-3PBA DF: 6 *trans*-DCCA DF: 100 P50: 0.93 P95: 4.28 *cis*-DCCA DF: 99 P50: 0.61 P95: 2.22 *cis*-DBCA DF: 97 P50: 0.60 P95: 3.85 CFCA DF: 30 P95: 0.27	*Cis*- and *trans*-DCCA were negatively associated with maternal education level and paternal education positively associated with DBCA (binary analyses)	Makris et al., 2022 [33]
Greece, Athens	Adults, (*n* = 40), part of multi-country study	2012–2014; Spot urine	Enzymatic hydrolyses; HPLC-MS-MS; QC: Isotope-labelled internal standards; Creatinine	LOQ: 0.003 DF: 100 P50: 0.50 Max: 6.6	4F-3PBA LOQ: 0.002 DF: 80 P50: 0.01 Max: 0.2 *trans*-DCCA LOQ: 0.002 DF: 100 P50: 0.6 Max: 4.0 *cis*-DCCA LOQ: 0.003 DF: 97.5 P50: 0.8 Max: 17.0 *cis*-DBCA LOQ: 0.019 DF: 50 P50: 0.02 Max: 6.0	NR	Li and Kannan 2018 [66]
Italy, NACII (PHIME)	Children 7 y (*n* = 199)	2014–2015; Spot urine	Enzymatic hydrolyses; UPLC-MS-MS; QC: Isotope-labelled internal standards and participation in G-EQUAS; Specific gravity	LOD: 0.018 DF: 81 P50: 0.56 Max: 36.0	4F-3PBA LOD: 0.019 DF: 24 Max: 1.3	No significant associations with population characteristics (education, age etc) or fish intake in binary analyses	Bravo et al., 2019 [67]
Italy, Rome	Adults (*n* = 55) patients referred to hospital for skin diseases	No information on sampling year; Spot urine	Acidic hydrolyses; GC-MS/SIM, QC: Internal standards; Creatinine	LOQ: 0.5 Males: DF: 34.5 Mean (SD): 0.52 (0.32) μg/g crea Females: DF: 65.5 Mean (SD): 0.74 (0.61) μg/g crea		Small sample size hamper significant results but tendencies to higher 3-PBA in samples collected in spring than winter, in females and among smokers, and if insecticides had been used inside or outdoor (binary analyses). Significantly associated with high intake of cooked vegetables	Fortes et al., 2013 [44]
Italy, Ragusa	Occupational, male greenhouse male workers (*n* = 30) exposed to alpha-cypermethrin and office workers as controls (*n* = 30)	No information on sampling year; Spot urine collected 3 months after occupational use of alpha-cypermethrin (Fastac)	Acidic hydrolysis; GS-MS; QC: no information; Creatinine	LOD: 0.04 Mean (SD): 7.8 (2.1) μg/g crea for workers <LOD for controls		Higher 3-PBA among workers occupationally exposed to alpha-cypermethrin	Costa et al., 2013 [50]
Italy, EPIC (substudy)	Adults (*n* = 69, 51 from Florence and 18 from Ragusa)	1993–1998; 24 h urine	Acidic hydrolysis; GC-MS; QC: Internal spiked standards; Creatinine	LOD: 2.5 nmol/L DF: 53.6 P50: 5.6 nmol/day Max: 52.8 nmol/day		Higher concentrations in Florence than Ragusa, tended to be higher in overweight/obese individuals	Saieva et al., 2004 [68]
Portugal, Oporto	Occupational, non-organic (*n* = 85) and organic (*n* = 36) farmers, controls from same area (*n* = 61)	No information on sampling year; Spot urine	No information on deconjugation; ELISA Immunoassay, QC: internal standards; Creatinine		Mean Total pyrethroid, μg/mmol crea, Organic: 0.06 Non-organic: 0.08 Controls: 0.13	No significant differences	Costa et al., 2014 [31]
Spain, INMA-Granada-Cohort	Male adolescents, 15–17 y (*n* = 134), 71.6% from urban area	2017–2019; FMV	No information on deconjugation; LC-MS/MS; QC: no information; Creatinine	LOD: 0.12 DF: 19.4 P95: 0.25		NR	Freire et al., 2021 [30]
Spain, Valencia Region, BIOVAL	Children, 5–12 y (*n* = 568), 78% from urban area	2016; FMV	Enzymatic hydrolyses; LC-MS/MS; QC: Isotope-labelled internal standards and participation in G-EQUAS; Creatinine	LOQ: 0.50 DF: 79 P50: 1.63 P95: 11.57	4F-3PBA LOQ: 0.13 DF: 4 DCCA LOQ: 5.00 DF: 20 P95: 46.7 *cis*-DBCA LOQ: 1.25 DF: 14 P95: 5.6	Intake of fresh vegetables within 72 h	Fernán-dez et al., 2020 [34]
Spain, Valencia	Women (*n* = 116), lactating mothers, 2–8 weeks after birth, 80% from urban areas	2015; FMV	Enzymatic hydrolyses; LC-MS/MS; QC: Isotope-labelled internal standards and participation in G-EQUAS; Creatinine	LOQ: 0.50 DF: 65 P50: 1.7 P95: 18.8		No significant associations with population characteristics or dietary variables	Fernán-dez et al., 2020 [35]
Spain, Catalonia and Galicia	Occupational, adults (*n* = 125), 36% farmworkers	No information on sampling year; Spot urine	Enzymatic hydrolyses; UPLC-MS-MS; QC: Isotope-labelled internal standards and participation in G-EQUAS; Specific gravity and creatinine	LOD: 0.018 DF: 82 P50: 1.5 Max: 20.5	4F-3PBA LOD: 0.019 DF: 54 P50: 0.08 Max: 0.34	Higher 3-PBA concentrations in farmworkers	Gari et al., 2018 [49]
Spain, Valencia	Children 6–11 y (*n* = 125)	2010; FMV	Enzymatic hydrolyses; LC-MS/MS; QC: Isotope-labelled internal standards; Creatinine	LOQ: 0.8 DF: 23 P95: 12.3 μg/g crea	4F-3PBA LOQ: 0.2 DF: 0 *trans*-DCCA LOQ: 0.4 DF: 26 P95: 4.44 μg/g crea *cis*-DCCA LOQ: 0.4 DF: 10 P95: 1.26 μg/g crea *cis*-DBCA LOQ: 0.8 DF: 23 P95: 3.77 μg/g crea	No significant associations with population characteristics or dietary variables	Roca et al., 2014 [69]

DF: detection frequency (%>LOD/LOQ); FMV: first morning void; German External Quality Assessment Scheme (G-EQUAS); NR: not reported; P50: 50th percentile (median); P25, P75, P90, P95: the respective percentile; GM: geometric mean; GSD: geometric standard derivation; SD: standard derivation; GW: gestational week; 3-PBA: 3-phenoxybenzoic acid; 4-F-3-PBA: 4-fluoro-3-phenoxybenzoic acid; *cis*-DCCA: *cis*-3-(2,2-dichlorovinyl)-2,2-dimethylcyclopropane-1-carboxylic acid; *trans*-DCCA: *trans*-3-(2,2-dichlorovinyl)-2,2-dimethylcyclopropane-1-carboxylic acid; *cis*-DBCA: *cis*-3-(2,2-dibromovinyl)-2,2-dimethylcyclopropane-1-carboxylic acid; CFCA (3-(-2-Chloro-3,3,3-trifluoroprop-1-enyl)-2,2- dimethyl-cyclopropane-carboxylic acid; crea: creatinine.

### 3.2. Chlorpyrifos

The specific metabolite, TCPy, of chlorpyrifos/chlorpyrifos-methyl was included in 23 studies representing 12 different countries (Table 3). Of these, the urine samples were collected among pregnant women in six studies, women after delivery in two studies, and children in seven studies. Only one study, among pregnant women in Norway (MoBa), was reported to be nationwide [70]. One study collected repeated spot urine samples in each trimester of pregnancy [71], one study collected a 24 h urine sample [68] while the remaining studies were based on single spot urine samples (14 studies) or FMVs (7 studies).

TCPy was analysed by LC-MS/MS in 16 studies and by GS-MS/MS in six studies while one study used Gas-Liquid Chromatography. The reported LODs/LOQs varied between the studies from a LOD of 0.02 µg/L to a LOQ of 0.8 µg/L. A deconjugation step based on enzymatic (16 studies) or acidic (4 studies) hydrolyses was described in all except for three studies. One of these, a study from Spain [72], reported a low detection frequency and maximum concentration compared to most other studies, and an underestimation of the concentration cannot be excluded. However, another Spanish study [71] reported an even lower detection frequency and maximum concentration despite inclusion of enzymatic hydrolyses. The two studies included different population groups, i.e., adolescent males and pregnant women, respectively, but sampling years (2017–2019 and 2016–2017) and LODs were comparable. Other studies from the Valencia Region in Spain from the same time period reported considerably higher detection frequencies and urine concentrations [34,35]. These results indicate regional differences in exposure levels but, as for the pyrethroids, the variation in urine sampling and analytical methods hamper direct comparison of the results across the studies. Overall, the TCPy concentrations varied considerably between the studies. The highest median concentrations were reported among children from Cyprus (6.72 μg/L) [33], adolescents and children from Belgium (4.45 and 3.87 μg/L) [36,37], and among adults from the Amirim community in Israel (4.32 μg/L) [73] based on urine samples collected between 2013 and 2018 (Table 3).

Only one study investigated time-trends in TCPy concentrations and reported an increasing trend from 2001 to 2017 among adolescents in Sweden with the highest median concentration in 2009 and the highest P95 concentration in 2017 [23]. Few of the studies investigated dietary exposure determinants and reported higher TCPy concentrations associated with high vegetable consumption [69,73,74] and negatively associated with organic food intake [73]. Besides, TCPy was associated with higher education level and lower BMI in some studies [35,39,41] which might reflect a diet with high vegetable and fruit content. Higher TCPy concentrations were also related to farm working [49]. None of the studies included urine samples collected after the ban of chlorpyrifos/chlorpyrifos-methyl in the EU in 2020.

**Table 3:** Human biomonitoring studies of chlorpyrifos/chlorpyrifos-methyl exposure based on urine samples from European populations.

Country, Region, (Cohort)	Study Population	Sampling Year and Method	Analytical Method and Quality Control (QC), Correction Method for Urine Dilution	LOD/LOQ, DF, and Urinary Concentrations (μg/L Unless Other Stated)	Exposure Determinants Reported	Ref
*Northern EU*				**TCPy**		
Denmark, Funen (OCC)	Pregnant women (*n* = 948)	2010–2012; Spot urine (morning after overnight fasting), GW 28	Enzymatic hydrolyses; LC-MS/MS; QC: isotope-labelled internal standards, participation in G-EQUAS; Creatinine	LOD: 0.3 DF: 90.4 P50: 1.61 P95: 8.49	TCPy associated with higher education level in binary analyses, no information on diet or home use of pesticides	Dalsager et al., 2019 [39]
Denmark, Funen, (DGCC)	Children, 10–16 y (*n* = 143)	2010–2012; Spot urine	Enzymatic hydrolyses; LC-MS/MS; QC: isotope-labelled internal standards, participation in G-EQUAS; Creatinine	LOD: 0.3 DF: 95.8 P50: 1.43 P95: 6.05	No significant associations with age, SES, urban or rural residence or sampling season	Andersen et al., 2021 [38]
Norway, MoBa	Pregnant women (*n* = 110, urine samples were combined into 10 pools, each comprising 1 mL urine sample from 11 women), nationwide	1999–2008; Spot urine	Acidic hydrolysis; GC-MS/MS; QC: Internal standards; Creatinine	LOD: 0.15 Mean: 2.33 GM (estimated): 0.99	NR	Ye et al., 2009 [70]
Sweden, Scania	Adolescents aged 17–21 y (*n* = approx. 200 per year in 2000, 2004, 2009, 2013, and 2017),	2000–2017; Spot urine	Enzymatic hydrolyses; LC-MS/MS; QC: Internal spiked standards, participation in Erlangen inter-laboratory comparison; Urine density and creatinine	LOD: 0.063 DF: 99–100 P50: 0.82–1.41 (max in 2009) P95: 3.50–6.54 (max in 2017)	Increasing temporal trend for TCPy with peak for median concentration in 2009 and indication of a downward trend thereafter	Noren et al., 2020 [23]
Sweden, Uppsala County (POPUP)	Women (*n* = 178), after delivery	2009–2014; Morning spot urine, 3 weeks after delivery	Enzymatic hydrolyses; LC-MS/MS; QC: Isotope-labelled internal standards, participate in Erlangen inter-laboratory comparison; Urine density	LOD: 0.02 DF: 100 P50: 1.32 Max: 14.2	NR	Gyllenhammar et al., 2017 [32]
Sweden, Värmland county (SELMA)	Pregnant women (*n* = 718)	2007–2010; FMV, GW 10	Enzymatic hydrolyses; LC-MS/MS; QC: Isotope-labelled internal standards, participate in Erlangen inter-laboratory comparison; Creatinine	LOD: 0.3035 DF: 100 GM (GSD): 1.25 (2.5)	NR	Tanner et al., 2020 [53]
*Western EU*						
Belgium, Flanders, FLEHS IV	Adolescents, 14–15 y (*n* = 415)	2017–2018; Spot urine	Enzymatic hydrolyses; LC-MS/MS; QC: Isotope-labelled internal standards; Specific gravity	LOD: 0.3 DF: 98.5 P50: 4.45 P95: 12.3 specific gravity normalised	NR	Schoeters et al., 2022 [36]
Belgium, Wallonia	Children, 9–12 y (*n* = 229), from five different locations (urban or agricultural)	2016; FMV	Enzymatic hydrolyses; GC-MS/MS; QC: Internal standards and materials from previous G-EQUAS programs; Creatinine	LOQ: 0.08 DF: 100 P50: 3.87 P95: 12.12	Negatively associated with intake of grey bread (graubrot)	Pirard et al., 2020 [37]
Germany, Mecklenburg-Vorpommern	Adults, 22–57 y (*n* = 50)	No information on sampling year; Spot urine	Acidic hydrolysis; GC-MS/MS; QC: Internal standards, Creatinine	LOD: 0.05 DF: 100 P50: 1.4 P95: 4.8	NR	Koch et al., 2001 [75]
Netherlands, Generation R	Pregnant women (*n* = 100)	2002–2006; Spot urine	Acidic hydrolysis; GC-MS/MS; QC: Internal standards; Creatinine	LOD: 0.15 DF: 100 P50: 1.2 P95: 6.4	Women with other ethnicity than Dutch had higher concentrations	Ye et al., 2008 [76]
*Eastern EU*						
Polen, Lodz,	Adult men, age <45 y (*n* = 315) recruited from fertility clinic	2008–2011; Spot urine	Enzymatic hydrolyses; GC-MS/MS; QC: Isotope-labelled internal standards; Creatinine	LOD: 0.50 DF: 100 P50: 1.14 P95: 7.99	NR	Dziewirska et al., 2019 [77]
Slovenia, Ljubljana (PHIME),	Children 7–8 y (*n* = 168) and their mothers (*n* = 168)	2016; Spot urine	Enzymatic hydrolyses; UPLC-MS-MS; QC: Isotope-labelled internal standards and participation in G-EQUAS; Specific gravity and creatinine	LOD: 0.02 Children: DF: 88 P50: 0.06 Max: 2.8 Mothers: DF: 84 P50: 0.18 Max: 4.5	Children had lower concentrations than mothers, women with normal BMI had higher concentrations than overweight women	Bravo et al., 2020 [41]
*Southern EU*						
Cyprus (ORGANIKO)	Children, 10–11 y (*n* = 177)	2017; FMV	No information on deconjugation; LC-MS/MS; QC: analysed in HBM4EU-accredited lab; Creatinine	LOQ: 0.8 DF: 100 P50: 6.72 P95: 13.7	Negatively associated with maternal education	Makris et al., 2022 [33]
Italy, NACII (PHIME)	Children, 7 y (*n* = 199)	2014–2015; Spot urine	Enzymatic hydrolyses; UPLC-MS-MS; QC: Isotope-labelled internal standards and participation in G-EQUAS; Specific gravity	LOD: 0.02 DF: 80 P50: 0.36 Max: 6.0	No associations with population characteristics, girls had higher concentrations than boys	Bravo et al., 2019 [67]
Italy, EPIC (substudy)	Adults (*n* = 69, 51 from Florence and 18 from Ragusa)	1993–1998; 24 h urine	Acidic hydrolysis; GLC-MS; QC: Internal spiked standards	LOD: 5.0 nmol/L DF: 78.3 P50: 29.5 nmol/day Max: 87.9 nmol/day	Highest concentration in Florence, no associations with other population characteristics	Saieva et al., 2004 [68]
Israel, IBS (nationwide) and Amirim community in Northern Israel	Adults from Amirim community with rural residence (*n* = 42) and Jewish participants from the Israel Biomonitoring Study (IBS) (*n* = 182)	2013–2014 (IBS in 2011); Spot urine	Enzymatic hydrolyses; GC-MS-MS; QC: Isotope-labelled internal standards and participation in G-EQUAS; Creatinine	LOD: 0.2 DF: 100 Amirim: P50: 4.32 P95: 21.8 IBS: P50: 2.34 P95: 8.52	Highest in the Amirim population and associated positively with vegetable intake and negatively with organic food intake.	Berman et al., 2016 [73]
Spain, Granada, INMA	Adolescent males, 15–17 y (*n* = 117)	2017–2019; FMV	No information on deconjugation; HPLC-MS/MS, QC: Internal standards; Creatinine	LOD: 0.039 DF: 34.2 Max: 1.21	NR	Suarez et al., 2021 [72]
Spain, Tarragona (EXHES-Spain)	Pregnant women (*n* = 54) from area of intense agricultural activity	2016–2017; Spot urine samples during each trimester (repeated samples)	Enzymatic hydrolyses, LC-MS/MS, QC: Isotope-labelled internal standards and participation in G-EQUAS;, Creatinine	LOD: 0.02 DF: 4 Max: 0.15	Not investigated due to low detection frequency	Bravo et al., 2020 [71]
Spain, Valencia Region, BIOVAL	Children, 5–12 y (*n* = 568), 78% from urban area	2016; FMV	Enzymatic hydrolyses; LC-MS/MS; QC: Isotope-labelled internal standards and participation in G-EQUAS; Creatinine	LOQ: 0.50 DF: 74 P50: 1.13 P95: 11.08	Lower if foreign birth country, differed between province of residence	Fernández et al., 2020 [34]
Spain, Valencia	Lactating mothers (*n* = 116), 2–8 weeks after birth, 80 % from urban areas	2015; FMV	Enzymatic hydrolyses; LC-MS/MS; QC: Isotope-labelled internal standards and participation in G-EQUAS; Creatinine	LOQ: 0.25 DF: 85 P50: 2.0 P95: 7.9	Associated with living near farming activities (<200 m). Negatively associated with pre-pregnancy BMI.	Fernandez et al., 2020 [35]
Spain, Valencia	Children 6–11 y (*n* = 125)	2010; FMV	Enzymatic hydrolyses; LC-MS/MS; QC: Isotope-labelled internal standards; Creatinine	LOQ: 0.8 DF: 86 P50: 3.40 μg/g crea P95: 12.97 μg/g crea	Positively associated with vegetable consumption.	Roca et al., 2014 [69]
Spain, Valencia, INMA	Pregnant women (*n* = 573)	2003–2006; Spot urine, GW 32	Hydrolysed urine (deconjugation method not described); UPLC-HRMS; QC: Internal spiked standards; Creatinine	LOQ: 0.8 DF: 39.1 GM: 0.49 Max: 117.3	Positively associated with vegetable consumption and sample season (highest in summer)	Llop et al., 2017 [74]
Spain, Catalonia and Galicia	Adults (*n* = 125) 36% farmworkers, occupational exposure	No information on sampling year; Spot urine	Enzymatic hydrolyses; UPLC-MS-MS; QC: Isotope-labelled internal standards and participation in G-EQUAS Specific gravity and creatinine	LOD: 0.02 DF: 95 P50: 3.2 Max: 20.0	Higher concentrations in farmworkers	Gari et al., 2018 [49]

DF: detection frequency (%>LOD/LOQ); FMV: first morning void; German External Quality Assessment Scheme (G-EQUAS); NR: not reported; P50: 50th percentile (median); P25, P75, P90, P95: the respective percentile; GM: geometric mean; GSD: geometric standard derivation; SD: standard derivation; GW: gestational week; TCPy: 3,5,6-trichloro-2-pyridinol; crea: creatinine.

### 3.3. Glyphosate

Glyphosate (Gly) and its main environmental degradation product aminomethylphosphonic acid (AMPA) are excreted unchanged in urine. Since, they do not undergo phase II conjugation a deconjugation step is not necessary for urine analyses [78]. Gly was analysed in 23 studies of which 15 studies also analysed AMPA. A single study analysed AMPA but not Gly (Table 4). Two studies collected single 24 h urine samples while single spot urine samples or FMVs were used in 12 and 8 studies, respectively. Three studies did not report the urine sampling method. LC-MS/MS and GC-MS/MS was equally used in 10 studies each for quantification of Gly and/or AMPA, while four studies used Enzyme-linked immunosorbent assay (ELISA) for quantification of Gly. LODs/LOQs varied between 0.05 and 1.0 µg/L. The studies were performed in 11 different EU-countries mainly from the western and southern part of the EU while two studies included samples from several countries.

In general, detection frequencies and reported urinary concentrations of Gly were considerably higher in studies using ELISA than those using LC- or GC-MS/MS and the results were not considered to be directly comparable. Most studies using LC- or GC-MS/MS to analyse samples from the general population had detection frequencies below 50% and therefor medians could not be obtained. Among these studies, the highest P95 concentrations for Gly were reported among children from Cyprus (1.01 µg/L) [33], lactating mothers from the Valencia Region in Spain (0.62 µg/L) [79] a nationally representative group of children from Germany (0.51µg/L) [80] and young children from Germany in a regional study (0.97 µg/L) [80]. Urine samples in these studies were collected between 2014 and 2017. In general, the majority of studies were based on urine samples collected after 2010 but one study from Germany included urine samples collected between 2001 and 2015 and reported a continuous increase in the fraction of samples with detectable concentrations with a peak in 2012–2013 [81].

A few studies found associations between urinary Gly and/or AMPA and higher intake of specific food items, e.g., beer and fruit juice [82], pulses and mushrooms [83], eggs and fruit [79], nuts and whole grain rice [84], and self-produced vegetables [85]. However, most of the included studies did not investigate or were unable to identify specific exposure determinants for the general population (Table 4). Two small studies from Ireland included occupational exposures among amenity horticultural workers and found higher urinary Gly-concentrations after work exposure with peak values 3 h after exposure [86,87].

**Table 4:** Human biomonitoring studies of glyphosate exposure based on urine samples from European populations.

Country, Region, (Cohort)	Study Population	Sampling Year and Method	Analytical Method and Quality Control (QC), Correction Method for Urine Dilution	LOD/LOQ, DF, and Urinary Concentrations (μg/L Unless Other Stated)		Exposure Determinants Reported	Ref
*Northern EU*				**Gly**	**AMPA**		
Denmark (DK-DEMOCOPHES)	Mothers (*n* = 13) and children 6–11 y (*n* = 14)	2011; Spot urine	ELISA immunoassay; QC: no information; Creatinine	LOD: 0.075 DF: 100 Mothers Mean:1.28 Max: 3.22 Children Mean: 1.96 Max: 3.31	NR	NR	Knudsen et al., 2017 [88]
Sweden, Scania	Young adults 18–19 y (*n* = 197)	2017; Spot urine	LC-MS/MS; QC: Isotope-labelled internal standards, participation in G-EQUAS for Gly; Creatinine and density	LOD: 0.1 DF: 20 P95: 0.24 (density adjusted)	LOD: 0.1 DF: 29 P95: 0.25 (density adjusted)	NR	Faniband et al., 2021 [89]
*Western EU*							
Belgium, Flanders, FLEHS IV	Adolescents, 14–15 years (*n* = 415)	2017–2018; Spot urine	GC–MS/MS; QC: internal standards and participation in G-EQUAS for Gly; Specific gravity	LOQ: 0.1 DF: 41.4 P95: 0.39 specific gravity normalised	LOQ: 0.1 DF: 55.9 P50: 0.11 P95: 0.37 specific gravity normalised	No significant associations with population characteristics	Schoeters et al., 2022 [36]
Belgium, Flanders, FLEHS III	Adults, 50–65 y (*n* = 181)	2012–2015; Spot urine	GC-MS-MS; QC: internal standards and participation in G-EQUAS for Gly; Specific gravity	LOQ: 0.1 DF: 42.5 P95: 0.31	LOQ: 0.1 DF: 58.6 P50: 0.10 P95: 0.40	NR	Cosemans et al., 2022 [90]
France, ELFE	Pregnant women (*n* = 1036), nationally representative	2011; Spot urine at delivery	UPLC-MS-MS; QC: internal standards, ISO/CEI certified labs, participate in inter-lab comparison; Creatinine	LOQ: 0.05 DF: 0.3	LOQ: 0.05 DF: 0.1	NR	Dereumeaux et al., 2016 [46]
France, Esterban (sub-sample)	Adults (*n* = 60) and children (*n* = 61) living close (within a radius of 500 m) or far from vineyards (*n* = 121)	2014–2016; FMV	LC-MS/MS; QC: no information; Creatinine	Glyphosate was not analysed	LOD: 0.02 Adults DF: 83 P50: 0.06 P95: 033 Children DF: 88 P50: 0.14 P95: 0.57	Higher AMPA if self-produced vegetables intake more than once per month (in adults), no other significant associations with population characteristics	Dereumeaux et al., 2022 [85]
France, 63 different districts	General population, median age of 53 y, (range: 0.5–94 y) (*n* = 6795),	2018–2020; Spot urine	ELISA immunoassay; QC: no information	LOQ: 0.075 DF: 99.8 Mean: 1.19 Max: 7.36 Values adjusted for BMI	NR	Higher in samples collected during spring/summer than winter, higher in males and children, among farmers, smokers, and individuals with high beer and fruit juice consumption, lower if filtered drinking water, analyses were adjusted for BMI but not for urine dilution	Grau et al., 2022 [82]
Germany, Baden-Württemberg	Adults, 18–70 y (*n* = 109)	2019; FMV	GC-MS-MS; QC: no information	LOD: 0.1 DF: 8	NR	After 10 days fasting none had Gly values above LOD	Grundler et al., 2021 [91]
Germany (GerES V)	Children/adolescents 3–17 y (*n* = 2144), nationally representative	2015–2017; FMV	GC–MS/MS; QC: internal standards and participation in G-EQUAS for Gly; Creatinine	LOQ: 0.1 DF: 52 P50: 0.1 P95: 0.51	LOQ: 0.1 DF: 46 P95: 0.48	Highest for medium SES, and children aged 1–13 y, no major exposure sources identified but higher for children living in larger communities	Lemke et al., 2021 [80]
Germany	Adults, ages 23–61 y (*n* = 41),	2016–2017; Spot urine	GC-MS/MS; QC: Isotope-labelled internal standards, Participating in G-EQUAS for Gly and HBM4EU inter-lab comparison for Gly and AMPA; Creatinine	LOQ: 0.05 DF: 66 P50: 0.09 Max: 0.33	LOQ: 0.05 DF: 90 P50: 0.20 Max: 2.54	NR—Method development study	Connolly et al., 2020 [92]
Germany	Children 2–6 y (*n* = 250), regional study	2014–2015; Spot urine and FMV	GC–MS/MS; QC: no information	LOQ: 0.1 DF: 63 Mean: 0.14 P95: 0.97	LOQ: 0.1 DF: 58 Mean: 0.13 P95: 0.44	NR	LANUV (2016), results presented in Lemke et al., 2021 [80]
Germany, GESB-Local sub-study in Greifswald	Young adults, aged 20–29 y (*n* = 399, approx. 40 per year in 2001, 2003, 2005, 2007, 2009 2011, 2012, 2013, 2014 and 2015)	2001–2015; 24 h samples	GC–MS/MS; QC: Isotope-labelled internal standards, Creatinine	LOQ: 0.1 For 2015 DF: 40 P95: 0.45	LOQ: 0.1 For 2015 DF: 42.5 P95: 0.38	Continuous increase in detectable fraction from 2001 with peak in 2012–2013, higher concentrations in males	Conrad et al., 2017 [81]
Germany, KarMeN study	Adults, 18–80 y (*n* = 301),	2012–2013; 24 h samples	LC-MS/MS; QC: Isotope-labelled internal standards	LOD: 0.05 DF: 30.9 P50 *: 0.11 Max: 1.36 * Among those with concentrations >LOD	LOD: 0.09 DF: 10.3 P50 *: 0.14 Max: 1.53 * Among those with concentrations >LOD	Associated with intake of pulses and for AMPA also mushrooms	Soukup et al., 2020 [83]
Ireland	Occupational, amenity horticultural workers (*n* = 20, repeated samples for different working tasks)	2016–2017; Spot urine	LC-MS/MS; QC: Isotope-labelled internal standards; Creatinine.	LOQ: 0.5 Pre-work samples (*n* = 29) DF: 62 GM: 0.68 P90: 2.99 Post -work samples (*n* = 28) DF: 82 GM: 1.17 P90: 4.95	NR	Higher after work exposure with peak values approx. 3 h after application	Connolly et al., 2018 and 2019 [86,93]
Ireland	Adults, 18–82 y (*n* = 50), pilot study	2017; FMV	LC-MS/MS; QC: Isotope labelled internal standards; Creatinine	LOQ: 0.5 DF: 20 Max: 1.35	NR	Small number of samples and no associations with dietary habits or lifestyle information obtained from questionnaires	Connolly et al., 2018 [94]
Ireland	Occupational, amenity horticultural workers (*n* = 17, repeated samples collected before and within 1 h after work task)	2015; Spot urine	LC-MS/MS; QC: Isotope-labelled internal standards; Creatinine	LOQ: 0.5 Pre-work samples (*n* = 31) DF: 35 GM: 0.42 max: 3.43 Post-work samples (*n* = 31) DF: 55 GM: 0.66 Max: 10.7	NR	Higher after work exposure	Connolly et al., 2017 [87]
UK, different locations in UK	Adults, 63.8 ± 10.4 y (*n* = 111 representing 65 twin pairs)	No information on sampling year or collection method	LC-MS-MS; QC: Isotope-labelled internal standards	LOD 0.05 DF: 53 P50: 0.05 Max: 2.8	LOD: 0.1 DF: 5.6 Max: 1.4	No difference between urban and rural residence	Mesnage et al., 2022 [60]
*Southern EU*							
Cyprus (ORGANIKO)	Children, 10–11 y (*n* = 177)	2017; FMV	GC-MS-MS; QC: HBM4EU-accredited lab (IPASUM), Creatinine	LOQ: 0.1 DF: 46 P95: 1.01	LOQ: 0.1 DF: 75 P50: 0.18 P95: 0.65	No significant associations with population characteristics	Makris et al., 2022 [33]
Portugal	Children 2–13 y (*n* = 41), four different regions	2018–2019; Spot urine	ELISA immunoassay; QC: no information	LOD: 0.6 DF: 95.1 Average: 1.77 Max: 4.35	NR	Increase with age, higher in girls, higher if living close (<1 km) to agricultural fields, use of agrochemicals in household, high consumption of home-produced food	Ferreira et al., 2021 [95]
Spain, Sevilla	Occupational, Female farmers (*n* = 20), indirect pesticide exposure,	No information on sampling year; FMV	UPLC-MS/MS; QC: Internal standards	LOQ: 1.0 DF: 5	LOQ: 0.5 DF: 0	Only one sample had detectable concentration (2 μg/L). Method validation study.	Martin-Reina et al., 2021 [96]
Spain, Valencia, BETTERMILK	Lactating mothers (*n* = 94), regional study	2015; FMV	LC-MS/MS; QC: Isotope-labelled internal standards, participation in G-EQUAS for Gly; Creatinine	LOQ: 0.1 DF: 54 P50: 0.12 P95: 0.62	LOQ: 0.1 DF: 60 P50: 0.13 P95: 0.69	Gly associated with high intake of eggs and fruit	Ruiz et al., 2021 [79]
*Eastern-EU*							
Slovenia	Children/adolescents 7–15 y (*n* = 246), agricultural region, two sampling periods (winter/summer)	2018; FMV	GC–MS/MS; QC: Internal standards; Creatinine and specific gravity	LOQ: 0.1 Winter DF: 27 P95: 0.19 Summer DF: 22 P95: 0.19	LOQ: 0.1 Winter DF: 50 P95: 0.29 Summer DF: 56 P50: 0.1 P95: 0.33	Higher detection rates related to lower age, and high intake of nuts and wholegrain rice	Stajnko et al., 2020 [84]
EU, countries not specified	Conventional (*n* = 99) and organic (*n* = 41) diet; healthy (*n* = 102) and chronically diseased (*n* = 199)	No information on sample collection	ELISA immunoassay; QC: some validation against GS-MS	data only reported in figures	NR	Higher if conventional than organic diet and in chronically ill than healthy subjects.	Krüger et al., 2014 [97]
EU, 18 different countries	182 urine samples (approx. 10 per country)	No information on sampling year or collection method	GC–MS/MS; QC: internal standards, Creatinine	LOQ: 0.15 DF: 44 Max: 1.56	LOQ: 0.15 DF: 36 Max: 2.63	Considerable regional variation, DF for Gly varied between 10 and 90% and for AMPA between 0 and 90%	Hoppe 2013 [98]

DF: detection frequency (%>LOD/LOQ); FMV: first morning void; German External Quality Assessment Scheme (G-EQUAS); NR: not reported; P50: 50th percentile (median); P25, P75, P90, P95: the respective percentile; GM: geometric mean; GSD: geometric standard derivation; SD: standard derivation; GW: gestational week; Gly: glyphosate; AMPA: aminomethylphosphonic acid; crea: creatinine.

## 4. Discussion

In this review, we identified HBM-studies that measured the internal exposure to pyrethroids, chlorpyrifos, and glyphosate in European population groups by analysing urinary concentrations of suitable biomarkers. We included studies that have been published from 2000 until June 2022. For all three substance groups the number of studies increased during the years and the majority were published during the last ten years. Variation in analytical methods displaying different sensitivities impacted the reported frequencies of detection and the urinary concentrations. Besides, the urine sampling methods varied, and the quantitative data was reported differently. Thus, direct comparison of the urinary concentrations across the studies was not always possible although many of the studies participated in external quality control programs such as the German External Quality Assessment Scheme (G-EQUAS). Further, less than half of the HBM4EU participating countries were covered and especially studies from the eastern part of Europe were scarce. Many studies were regional with relatively small sample size. Despite these shortcomings, the results indicate a widespread exposure to these substances in the general EU population with marked geographical differences. Studies form Cyprus and the Valencia region in Spain reported the highest urinary concentrations for all the included pesticides. Thus, identification of the main exposure sources in these areas can be used to reduce exposure. In general, children had higher urinary concentrations of the pesticide metabolites than adults as also seen in studies from, e.g., the US [25]. An obvious explanation is a relatively higher food intake per kg body weight in children leading to higher exposure levels from pesticide residues in food, but also other physiological and behavioural differences may predispose children to elevated exposure [6,99,100].

The organophosphate chlorpyrifos was for decades one of the most widely used insecticides in agriculture worldwide [101] leading to a widespread exposure of the general population from residues in food, as reflected in the high detection frequency of TCPy in most of the included studies. However, the authorization for chlorpyrifos (and chlorpyrifos-methyl) in the EU was withdrawn by February 2020 because of concern for genotoxicity and developmental neurotoxicity [102]. Before this ban, acceptable daily intake (ADI) for chlorpyrifos was reduced from 0.01 to 0.001 mg/kg body weight/day in 2014. Because of parallel reductions in EU maximum residue levels (MRLs) in food items, the exposure level in the general population would be expected to have decreased in this period. A corresponding drop in urinary TCPy concentrations could not be documented from the included studies, but no urine samples collected after the ban in 2020 were included. Thus, the concentrations reported in the current studies can be used for comparison in future studies.

The studies on pyrethroids showed higher urinary concentrations of 3-PBA in samples collected in the most recent years indicating an increasing population exposure to these insecticides. A rise in exposure would be expected, since pyrethroids have replaced organophosphate insecticides in biocidal products and to some degree also as plant protection products. Accordingly, increasing urinary metabolite concentrations were also found in the human biomonitoring programs The National Health and Nutrition Examination Survey (NHANES) in the US [103] and the Canadian Health Measures Survey (CHMS) [104]. Indoor use of pyrethroids were associated with higher urinary metabolite concentrations in several of the included studies as also demonstrated in studies form the US [6,105].

Glyphosate is the most used pesticide worldwide and also one of the most widely used herbicides in agriculture in the EU [106] but HBM-data are limited, both from Europe and elsewhere. The included studies indicate a widespread low Gly exposure with rather low detection frequency in urine probably reflecting the low urinary excretion fraction of 1% for Gly estimated in humans after oral exposure [8,89]. In general, the knowledge on toxicokinetics of Gly and AMPA in humans is limited and more information on, e.g., uptake after inhalation exposure is needed. 

For both pyrethroids and glyphosate there is a paucity of HBM-data regarding exposure levels in potentially higher exposed (sub)populations in occupational and environmental settings, e.g., studies focusing on exposure after indoor and outdoor residential use, exposure from living in vicinity to pesticide treated areas, occupational and para-occupational exposure levels including take-home exposure after work. Such studies are complicated by the fact that currently used pesticides are rapidly metabolised and excreted within few days and therefor urinary concentrations reflect only recent exposure to the specific pesticides. Therefore, such studies require careful planning to obtain valid information on exposure levels and peak exposures.

The results from this review illustrate the need for harmonisation of the analytical methods as well as the reporting of HBM-data to enable comparisons of exposure levels across studies to obtain information on population differences in exposure sources and time-trends. Such a process was initiated within the HBM4EU initiative, in which harmonised HBM-data was obtained for the prioritised pesticides either by analysing new urine samples or by quality-assurance of data already collected in the HBM4EU Aligned Studies [13,107]. In this way, HBM-data on glyphosate was achieved among children from five countries, Slovenia, Germany, France, Belgium and Cyprus [16], and adults from Germany, Switzerland, France, and Iceland [15]. Data on pyrethroids and chlorpyrifos were obtained among children from Slovenia, Cyprus, France, Belgium, the Netherlands, and Israel and among adults from Germany, France, Switzerland and Israel [14,17,18]. These HBM-data from the HBM4EU Aligned Studies is available for visualization in the EU-HBM dashboard: https://www.hbm4eu.eu/what-we-do/european-hbm-platform/eu-hbm-dashboard/ (accessed on 18 10 2022) along with HBM-data from some of the studies included in this review. However, more HBM-data are needed to get an EU-wide picture of the exposure and to evaluate differences between countries and population groups, time trends, and age-related differences in exposure levels and sources. The data presented in this review, combined with the HBM-data from the HBM4EU-aligned studies, can be used as a baseline for future studies of exposure to these pesticides. Furthermore, there is a need to establish harmonised and sensitive biomarkers for other frequently used pesticides/pesticide groups in order to get an overall picture of pesticide exposure in Europe.

## Figures and Tables

**Table 1:** Urinary biomarkers for pyrethroids, chlorpyrifos, and glyphosate.

Biomarker	Metabolite	Parent Pesticide
3-PBA	3-phenoxybenzoic acid	generic metabolite of most pyrethroids, e.g., cypermethrin, deltamethrin, permethrin, lambda-cyhalothrin, etofenprox, tau-fluvalinate, esfenvalerate, fenpropathrin, (but not cyfluthrin or bifenthrin)
4-F-3PBA	4-fluoro-3-phenoxybenzoic acid	cyfluthrin
*cis*-DCCA	cis-3-(2,2-dichlorovinyl)-2,2-dimethylcyclopropane-1-carboxylic acid	*cis*-permethrin, *cis*-cypermethrin, cyfluthrin
*trans*-DCCA	trans-3-(2,2-dichlorovinyl)-2,2-dimethylcyclopropane-1-carboxylic acid	*trans*-permethrin, *trans*-cypermethrin, cyfluthrin
*cis*-DBCA	cis-3-(2,2-dibromovinyl)-2,2-dimethylcyclopropane-1-carboxylic acid	deltamethrin
CFCA	3-(-2-Chloro-3,3,3-trifluoroprop-1-enyl)-2,2- dimethyl-cyclopropane-carboxylic acid	bifenthrin, λ-cyhalothrin and tefluthrin
TCPy	3,5,6-trichloro-2-pyridinol	chlorpyrifos and chlorpyrifos-methyl
Gly	glyphosate	glyphosate
AMPA	aminomethylphosphonic acid	glyphosate, main environmental biodegradation product of glyphosate

## Data Availability

Not applicable.
